# Historical Range Contraction and Extent of Harbour Porpoises (*Phocoena phocoena*) in the Baltic Sea Revealed by Archival Newspapers

**DOI:** 10.1002/ece3.73475

**Published:** 2026-05-11

**Authors:** M. Aiken, P. Erikson, C. Kinze, J. Carlström

**Affiliations:** ^1^ Department of Population Analysis and Monitoring Swedish Museum of Natural History Stockholm Sweden; ^2^ Department of Anthropology Trent University Peterborough Canada; ^3^ Cetacean Atlas of Denmark Frederiksberg Denmark; ^4^ Natural History Museum of Denmark University of Copenhagen Copenhagen Denmark; ^5^ Department of Biology Lund University Lund Sweden

## Abstract

Over the past several centuries, the Baltic Proper harbour porpoise ( *Phocoena phocoena* ) has undergone significant population declines, resulting in its current IUCN classification as critically endangered. While conservation efforts are extensive and multinational, the population's abundance has only been estimated once (SAMBAH Project, 2011–2013). Ad hoc historical sources and the sub‐fossil archaeological record, dating to 7000 years BP, suggest that the population once had a wider distribution in the Baltic Sea. However, the historical abundance and distribution of the Baltic Proper harbour porpoise population remain largely unknown, especially before the mid‐20th century. This study examines archival Swedish newspaper records from the late 1700s to the early 1900s to assess the presence and distribution of porpoises. Digitized articles were searched for by keyword in the National Library of Sweden Newspaper Archive. This dataset was combined with HELCOM/ASCOBANS historic data. The records show that harbour porpoises historically occurred regularly along the entire Swedish coast, including the northernmost parts of the Gulf of Bothnia, where the species is virtually absent today, suggesting a notable range retraction. While harbour porpoises appeared less frequently in the Baltic Sea than along the west coast of Sweden, the number of Baltic records indicates that porpoises occurred more frequently than today. The peak occurrence of porpoises during spring and summer suggests that the Gulf of Bothnia historically constituted an important foraging habitat for animals migrating from the southern Baltic. By integrating historical records with modern conservation data, this study provides critical insights into the long‐term effects of human activity on the Baltic Proper harbour porpoise. Understanding past ranges and approximate population size is vital for guiding more effective conservation strategies.

## Graphical abstract

Archival Swedish newspapers from the 18th to 20th centuries reveal that the Baltic harbour porpoise once utilized the waters along the full Swedish coast, reaching far north into the Gulf of Bothnia, areas where it no longer occurs today. These records show a major range loss and highlight the importance of historical baselines for conservation.

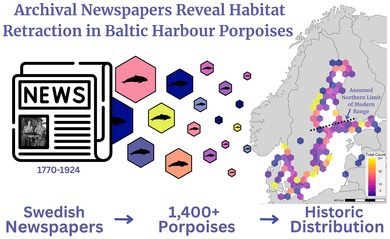

## 1 Introduction

Baltic Proper harbour porpoises ( *Phocoena phocoena* ) are listed as critically endangered by the IUCN (Carlström et al. 2023) and the Baltic Marine Environment Protection Commission (HELCOM 2025), with an extant population of ~500 individuals (Amundin et al. 2022). While research and monitoring initiatives are extensive and multinational (Benke et al. 2014; Carlén et al. 2018; Döhring 2022; Koschinski et al. 2024), the population's abundance has only been estimated once, about a decade ago (SAMBAH Project, 2011–2013; Carlén et al. 2018; Amundin et al. 2022). Although archaeological evidence suggests that the harbour porpoise was once more widespread in the southern and central Baltic region (Sommer et al. 2008; Lõugas and Bērziņš 2023; van den Hurk and Aiken 2025), there is no reliable information regarding the historic range and presence. Here, we use a marine historical ecology (MHE) approach, examining records of harbour porpoises from archival Swedish newspapers and Baltic region documents from the late 1700s to the early 1900s to assess the presence and distribution of harbour porpoises in the Baltic region.

MHE offers valuable insights into the conservation of modern ecosystems by combining historical perspectives with contemporary ecological practices (Al‐Abdulrazzak et al. 2012; Kittinger et al. 2014; Engelhard et al. 2016). This interdisciplinary approach integrates previously disparate fields, including ecology, history, archaeology, economics, and fisheries science, to enhance understanding of long‐term human‐ocean interactions, informing effective management strategies for marine environments. By placing modern studies into context through a historical perspective, MHE combats the often deleterious effects of shifting baseline theory (Pauly 1995; Keegan 2009; Orton 2016; Atmore et al. 2021), providing evidence regarding the drivers of past change (Lotze and Worm 2009). Through the development of historical baselines and ranges, MHE has aimed to understand the effects of anthropogenic disturbances on marine ecosystems, including their structure and function (Engelhard et al. 2016; Thurstan 2022).

Historic newspapers serve as valuable resources for examining past faunal distributions by reporting sightings and observations of animals. These archives have been used to gather data on species presence (for example: urban biodiversity, Vuorisalo et al. 2001; cetaceans, Brito and Sousa 2011; commercial fisheries, Herbst et al. 2023), relative abundance (for example: lake sturgeon, Cochran and Elliott 2012), seasonal behaviors (for example: whale occurrence, Brown and Wiedenmann 2024), and human‐animal interactions (for example: coastal ecosystems, Sandoval Gallardo et al. 2021), revealing patterns that contribute to our understanding of historical biodiversity. This does not come without difficulties: the interpretation of historic newspapers can present challenges in understanding inconsistencies and variability in how natural phenomena were reported, as they often reflect local, regional, and national interests, without a focus on scientific accuracy. The reports are often disparate, requiring researchers to manually filter through records (van Erp et al. 2018). Moreover, descriptions can be vague or imprecise, making it difficult to definitively identify species. However, the benefits are considerable. In Sweden, historic newspaper accounts offer valuable snapshots of the environment from periods when natural history was becoming a more prominent interest, with growing public fascination with documenting and understanding wildlife (Hodacs 2010, 2011). Although the accounts may lack the precision of modern scientific observations, they provide a unique resource to track the presence and distribution of species that would otherwise be inaccessible (Ferretti et al. 2014).

In this study, we use historical newspaper records from Sweden to reconstruct the past presence, spatial distribution, and relative occurrence of harbour porpoises in the Baltic Sea region. Our aim is not to derive quantitative estimates of historical population size or to directly test causal mechanisms underlying long‐term change, as the opportunistic and heterogeneous nature of historical newspaper reporting prohibits such inference. Instead, we treat these records as qualitative‐to‐semi‐quantitative evidence of where and when harbour porpoises were encountered, including observations of calving and juvenile animals, which provide insight into reproductive activity and population structure. These historical observations provide a baseline against which contemporary distribution and population size can be contextualized. When combined with present‐day observational data, they allow us to assess long‐term changes in distributional extent and to generate testable hypotheses about the ecological and anthropogenic processes that may have contributed to the species' decline in the Baltic Sea.

## 2 Methods

**FIGURE C1:**
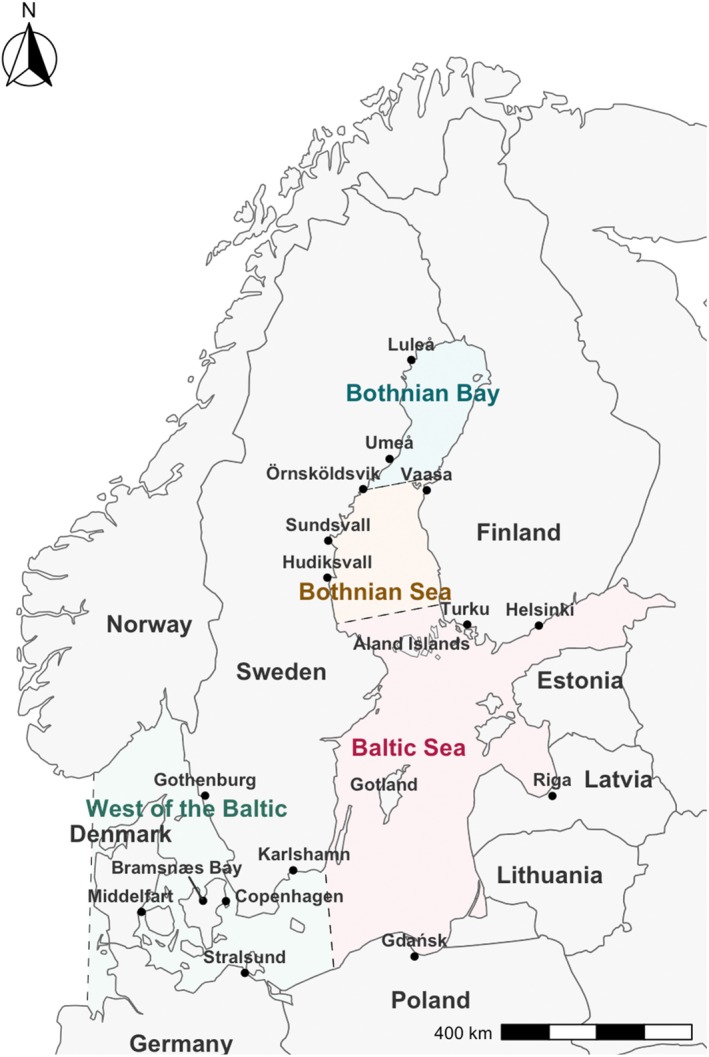
Map of the Baltic region with countries, key cities and islands, and division of subregions.

Reports of harbour porpoises in the Baltic and Belt Sea were obtained from Swedish Archival newspapers digitized and held by the National Library of Sweden (https://tidningar.kb.se/). Into the early 1900s, newspapers were primarily general‐interest publications, a style referred to as miscellaneity, the intentional combination of diverse, unrelated, and often short‐form content within a single publication (Turner 2020). The newspaper database was searched by year, using key terms from the earliest records in the archive, from 1645 to 1924 (newspapers published after 1924 were not publicly available due to copyright law). The terms included: phocoena, phocaena, phocana, tumlare (Swedish for “porpoise”), marsvin (alternative Swedish name for “porpoise”), communis (a binomial nomenclature used “Phocoena communis” for harbour porpoises), nisa (Norwegian for harbour porpoise), delphinus (“ *Delphinus phocoena* ”), sjöodjur (Swedish for “sea monster”), pyöriäinen (Finnish for “porpoise”), and valfisk (Swedish for “whale”; see Appendix A for more detail on search terms). Hits from the search terms were then manually assessed to identify mentions of harbour porpoises. This excluded information about harbour porpoises in other bodies of water (i.e., in accounts of travel across the Atlantic), mentions in serialized fiction, and accounts that were not harbour porpoises (based on size or other physical characteristics). From the mentions of harbour porpoises in the Baltic and the Belt Sea (Figure C1) waters, specific information was recorded: the name of the newspaper, the search term used, the year, the date of publication, the most exact date (i.e., if the article mentioned the date of observation or catch either as a numerical date or more colloquially, for example: “last Friday”), the toponym associated with the mention, the coordinates associated with that toponym (estimated from the description, for example: “off the coast of Umeå”), and a translation of the report into English. Each mention was given a “Report ID” (RID) to identify the report. A map showing the locations of key cities, sub‐regional divisions, and relevant sea areas is provided in Appendix C (Figure C1). This map is used throughout the Results and Discussion to contextualize observations.

The RIDs were collated into Observation IDs (OID; Table S1), combining observations that were reported multiple times. This included the most exact date available, toponym, coordinates, and a combined version of the published stories. From the newspaper stories, the following information was collected for each OID: the number of reported harbour porpoises (using two if multiple porpoises were seen, but no number was given), the gear involved (i.e., gun, fishing net, etc.), and biological information about the porpoise(s) (length, weight, girth, sex, and information about pregnancy or calves).

The archival Swedish newspaper dataset was supplemented with records from the HELCOM/ASCOBANS Harbour Porpoise Database (HELCOM 2023). To ensure comparability, the HELCOM/ASCOBANS data were first restricted to records dated up to 1924, matching the temporal coverage of the Swedish archival material. This cropped dataset, containing records originating from the Finnish Ministry of the Environment and Gauja National Park, was then cross‐checked against the Swedish dataset to identify and remove any duplicate observations. After this filtering and de‐duplication process, 123 additional observations, representing a minimum of 189 porpoises not present in the Swedish archival dataset, were added to the final compiled dataset.

**FIGURE A1:**
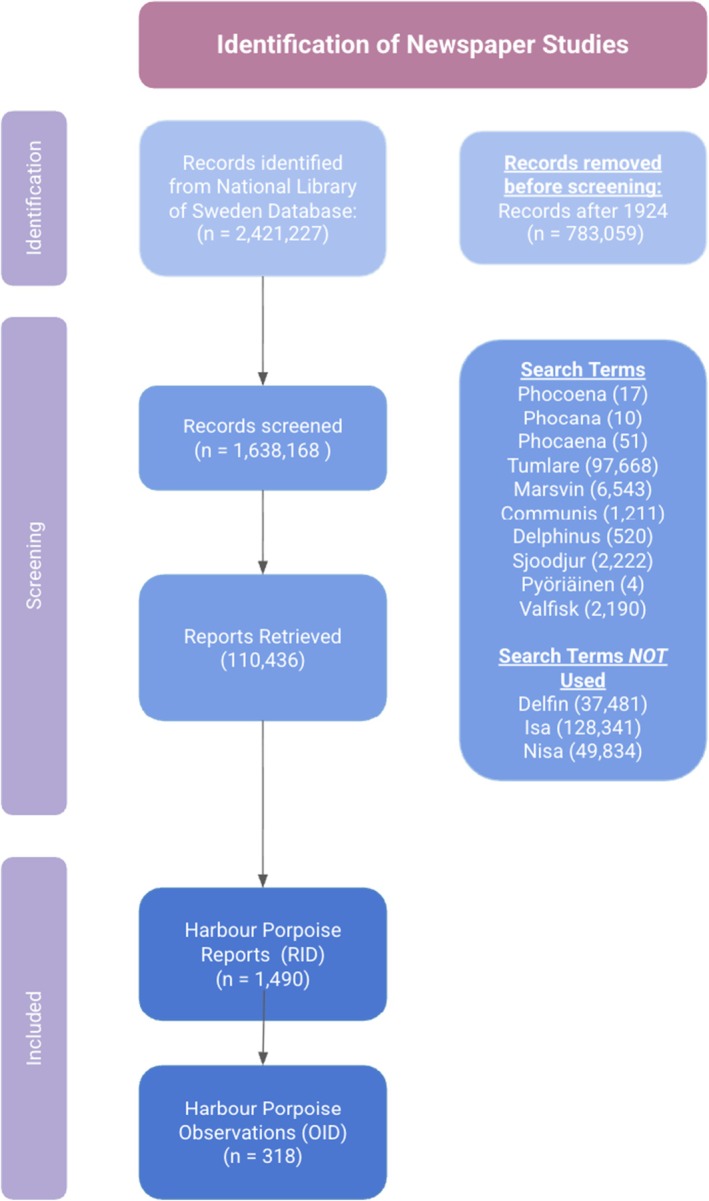
Flow chart showing the processes of identifying newspaper reports of harbour porpoises.

The dataset produced represents reported occurrences rather than absolute abundance. Each record reflects a series of filtering steps from biological reality (absolute abundance) to human observation to reporting in a newspaper to archiving, and finally, to inclusion in the dataset (Figure A1). Consequently, the number of porpoises identified by this study should be treated as a minimum. The reports may disproportionately reflect unusual observations, for example, the larger harbour porpoise population on the west coast of Sweden may be less widely reported than a smaller more uncommon population in the Baltic Proper. Despite these limitations, the minimum counts are expected to show broad historic patterns of presence and distribution currently unknown.

### 2.1 Data Analysis

Data analysis was conducted in R version 2024.12.1 + 563 (R Core Team 2022). To avoid the inclusion of non‐porpoise cetaceans that were incorrectly identified as porpoises in the newspapers and not excluded during data collection, a set of exclusion criteria based on size was developed. This included upper limits on weight (100 kg) and length (2 m) based on maximum sizes of harbour porpoises and the lower averages for other species known to visit the Baltic region, including the common dolphin ( *Delphinus delphis* ), bottlenose dolphin ( *Tursiops truncatus* ), white‐beaked dolphin ( *Lagenorhynchus albirostris* ), and newborn orcas ( *Orcinus orca* ; Table B1). The exclusion criteria identified seven (~0.6%) animals as non‐porpoise (OID: 10, 101, 118, 157, 230, 281, and 292). Additionally, OID 4 was identified as a bottlenose dolphin mentioned in other historic records. These animals were excluded from the downstream analysis.

The location and number of porpoises observed were visualized by OID in R using hexbin maps with a bin width of 75 km. To examine any correlation between the number of newspapers published and the number of observations over time, several different tests were carried out. The normality of the OID data and the number of archived newspapers were tested using the Shapiro–Wilk normality test, which showed that the data were not normally distributed. Spearman's test was used to examine the correlation between the number of OIDs and the number of newspapers published, as well as the number of porpoises observed and the number of newspapers published. Poisson generalized linear models (GLM) were used with a log link function to examine the effects of newspaper counts and time period on the number of porpoises reported by year. To allow for non‐linear relationships, general additive models (GAM) were also fitted with the annual number of reported harbour porpoises as the response variable and the annual number of archived newspapers as the predictor. Since the response variable consisted of count data, the model assumed a Poisson error distribution with a log link function. Newspaper availability was included as a smooth term using a penalized thin‐plate regression spline, with smoothing parameters selected automatically using generalized cross‐validation. To assess if harbour porpoises were reported more frequently after 1900, as suggested anecdotally in newspaper reports, years were grouped into pre‐1900 and post‐1900 periods. Differences in porpoise counts between these periods were first evaluated using a non‐parametric Mann–Whitney U test. In addition, a Poisson GLM including both time period and newspaper availability was fitted to account for differences in the number of newspapers archived. To examine spatial heterogeneity, separate Poisson GLMs were run for each sub‐region (Bothnian Bay, Bothnian Sea, Baltic Sea, and west of the Baltic), using the same model structure. Newspaper availability was aggregated across the sub‐regions, as historical newspapers frequently reprinted observations from outside their sub‐region of publication, preventing a reliable assignment of newspaper effort to specific sub‐region.

The number of OIDs was visualized geographically, using hexbins, as well as temporally in a timeseries plot (Figure 4). The annual number of newspapers archived by the National Library of Sweden over the time period was extracted from the database and included. To examine the seasonality of the number of observed harbour porpoises by sub‐region, the monthly number was summarized by the four sub‐regions: West of the Baltic Sea, the Baltic Sea, the Bothnian Sea, and the Bothnian Bay. The delineation between the West of the Baltic Sea and the Baltic Sea is based roughly on the management border suggested by Carlén et al. (2018); here, longitude 16*°* E is used (Figure C1). The delineation between the Baltic and Bothnian Sea is just above the Åland Islands (latitude 60.75*°* N), and the delineation between the Bothnian Sea and Bothnian Bay is at Vasa, Finland (latitude 63.17*°* N). A time series plot was made to visualize the number of harbour porpoises observed over time, and seasonality of harbour porpoise locations was examined by plotting the number of porpoises observed by month and by sub‐region, including the West of the Baltic, Baltic Sea, Bothnian Sea, and Bothnian Bay. For this purpose, data from all years were combined, enabling a broader view of seasonal trends without year‐specific bias.

Two calves were identified based on newspaper reports (OID: 109, 214). Additionally, animals were assigned to an age class (neonate, calf, juvenile, or adult) based on length (m) following the four‐class criteria established by Lockyer (1995) and van Elk et al. (2019), as detailed by Neimanis et al. (2022). Since sex information was unavailable for the historic dataset, sex‐specific differences in maturation length required an adaptation of the criteria (Table B2). Specifically, the cutoff for the adult class was set at 1.29 m. This length corresponds to the minimum published length for maturity in modern male harbour porpoises and is lower than that for female harbour porpoises (1.39 m). The 1.29 m length was selected to ensure that all individuals considered mature in the modern population (both male and female) were included in the historic “adult” category. While inclusive of all adults, the 1.29 m cutoff likely results in the inclusion of some larger juvenile females within the historic “adult” class.

Information on porpoise length was included in the descriptions for ~12% (*n* = 176) of the total number of harbour porpoises, weight was included for ~20% (*n* = 79), and girth for ~0.4% (*n* = 6). These were standardized to the metric system to allow comparison. Several measurement systems were recorded in the newspapers. In addition to the metric system, traditional Swedish units of measurement, which were standardized in 1665, were also used, including aln (ell; pl. alnar): from 1605 to 1863 59.38 cm, from 1863 onward 59.37 cm; famn (fathom): 3 alnar; fot (foot): ½ aln. Mass measurements included skålpund: 0.42507 kg, and lispund: 8.502 kg. Individuals were excluded if both measurements were included in the newspaper report, but only one met the criteria (ex., weight: 17 kg and length: 2 m would not count as a juvenile). The geographic location and the number of juvenile porpoises over time were plotted. The length of the porpoises is plotted over time, separating the five age classes.

## 3 Results

The first records of harbour porpoises in Swedish newspapers are from May 1772 and were published by the newspaper “Hwad Nytt?” (What's New?). Reports of porpoises in Swedish newspapers did not become regular, with yearly mentions, until the 1880s. In total, between 1772 and 1925, harbour porpoises were reported 1490 times in Swedish archival newspapers, resulting in 318 unique OIDs (Table S1). The reports indicate a minimum of 1266 harbour porpoises observed. After supplementing the Swedish archival dataset with the 123 unique observations from the historic HELCOM/ASCOBANS dataset, there are a total of 441 observations accounting for at least 1455 harbour porpoises.

### 3.1 Distribution and Occurrence

**FIGURE 1:**
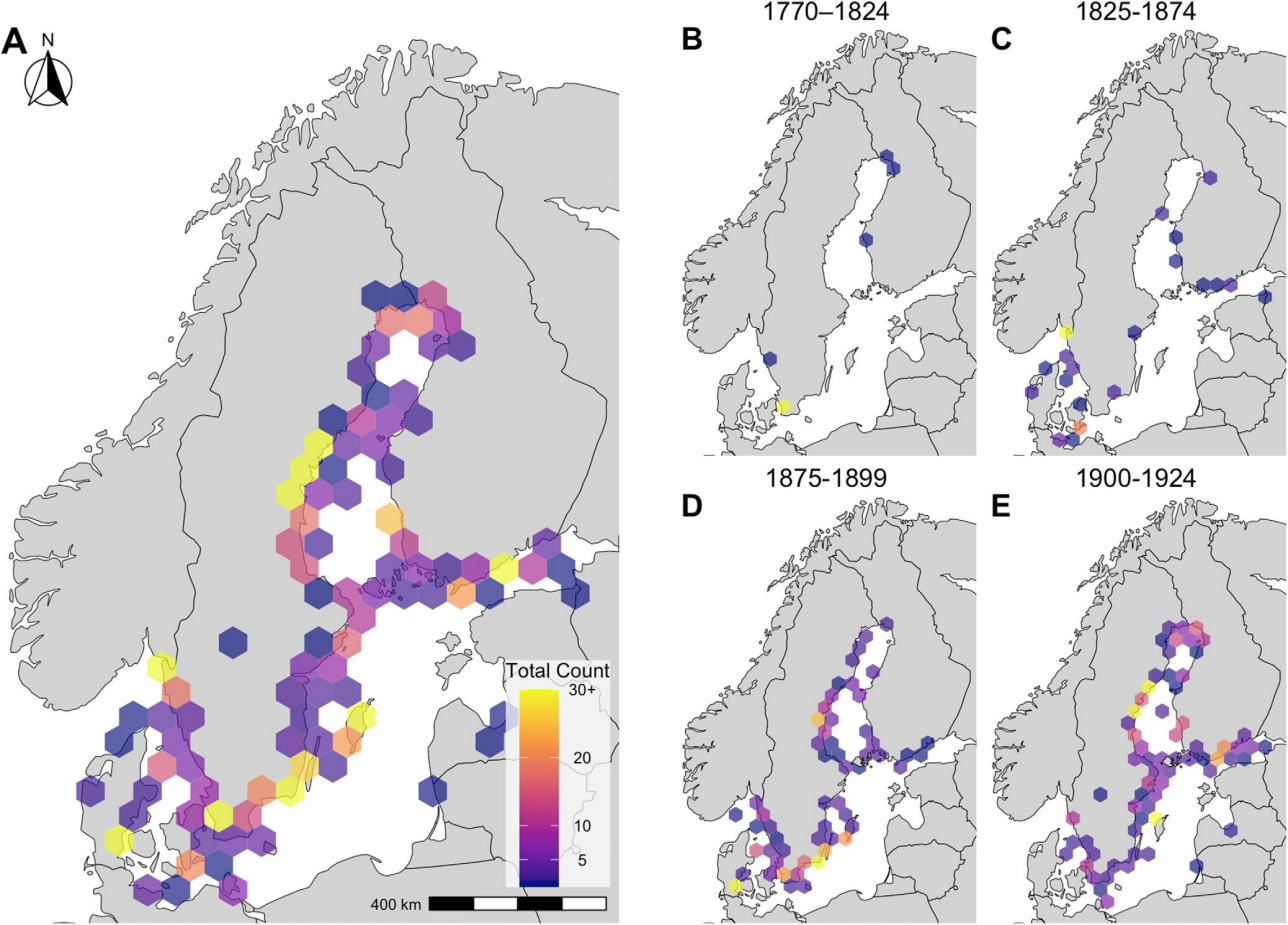
Hexplot of all harbour porpoises recorded in Swedish newspapers and the HELCOM/ASCOBANS dataset (HELCOM 2023) from (A) 1772 to 1924, (B) 1770 to 1824, (C) 1825–1874, (D) 1875–1989, and (E) 1900–1924. The weight (color) is based on the number of porpoises.

**FIGURE 2:**
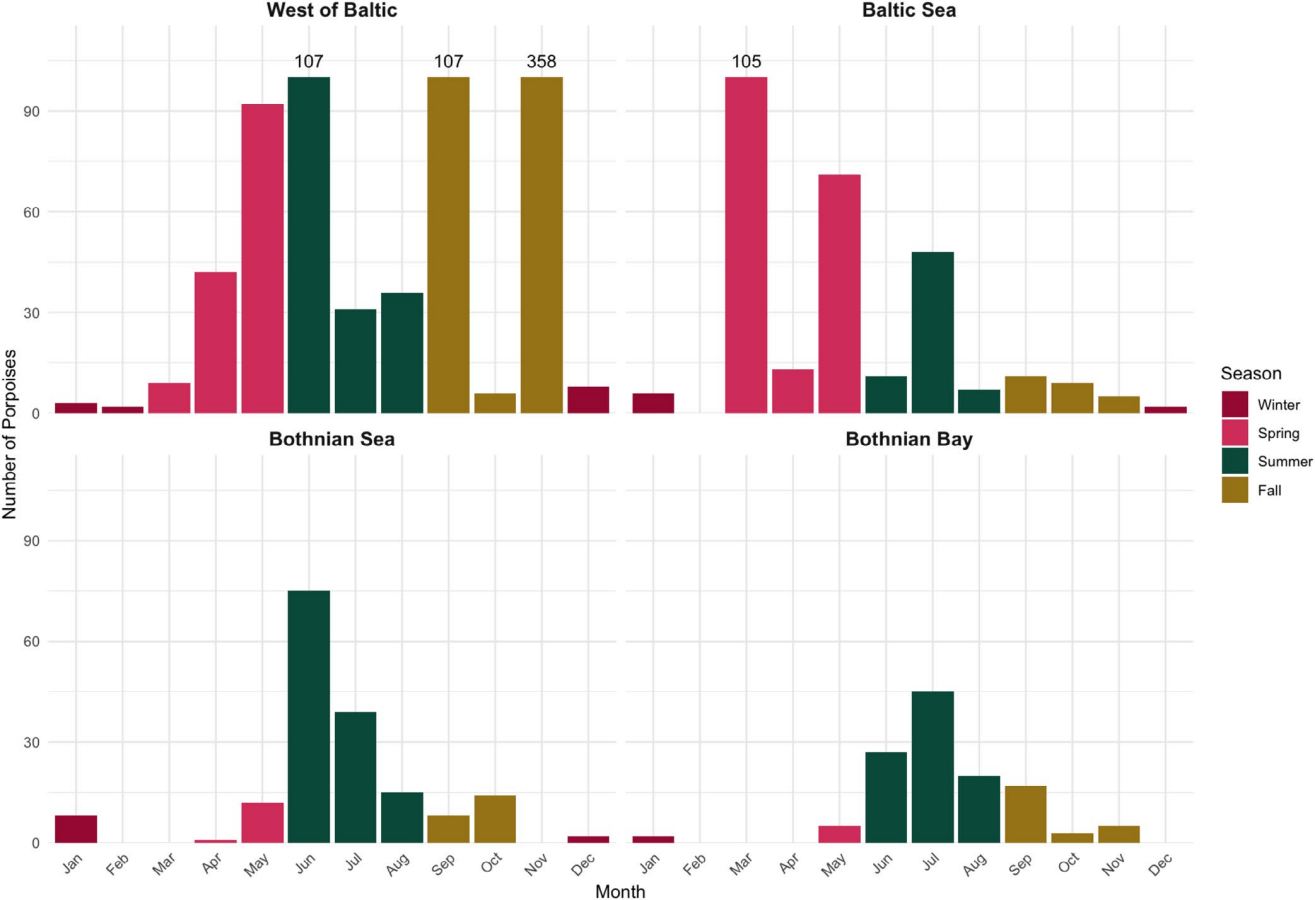
Plots showing the number of observed harbour porpoises by sub‐region and month. The colors are based on seasons, and the number of porpoises is separated by month. The figure has an upper limit of 100 recorded porpoises per month to better visualize the trends. For truncated bars, the total number of observed porpoises is shown above the bar.

**FIGURE C2:**
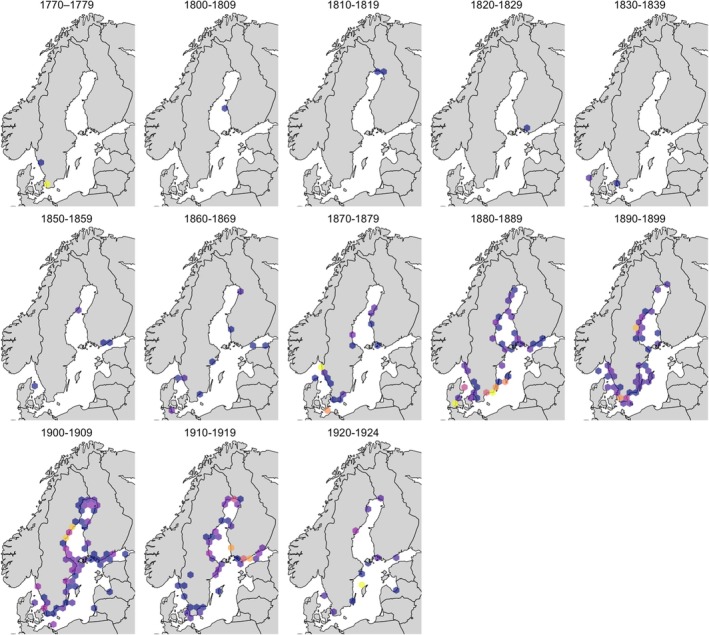
Hexplot of all harbour porpoises recorded in Swedish newspapers and the HELCOM/ASCOBANS dataset (HELCOM 2023) visualized in 10‐year increments. The weight (color) is based on the number of porpoises.

The combined dataset shows that from 1772 to 1924, harbour porpoises were observed along the entire Swedish and most of the Finnish coast, as well as in Danish, German, Lithuanian, and Latvian waters (Figure 1, Figure C2; see Figure C1 for city and sea locations). The highest historic number is reported from the Little Belt Sea in Denmark, where, during the hunting season in November 1880, 800 harbour porpoises were reported to have been killed. Specifically, from the 27th to 30th November 1880, 349 harbour porpoises were caught over these three days (OID: 31; see Kinze 2008). Other hotspots include Kattegat in southern Sweden, where a 1775 report mentions the presence of over 100 porpoises in the Kattegat “accompanying” the herring (OID: 2), and Northern Gotland in the Baltic Sea, where, in addition to the porpoises regularly caught, 100 porpoises are reported as observed and killed by drift ice in March 1924 (OID: 324). The Bothnian Bay sub‐region around Sundsvall also has a high number of porpoises reported (*n* = 120), but these are the results of a large number of individual reports (31 OIDs), rather than a single larger event. Örnsköldsvik has the highest number of OIDs within a 50 km radius, 36, with a relatively low number of porpoises observed per OID, with a total of 52 porpoises. Luleå is similar, with the sixth highest number of OIDs, 12, but the number of porpoises is only 20. The seasonality of the harbour porpoises' location shows that they were less present in the Bothnian Bay and Bothnian Sea in the winter months, with no observations from February through April and few animals in December and January (Figure 2). In the Baltic Sea, a higher number of reports occurs in all seasons but autumn. West of the Baltic Sea, the highest number of animals is reported during the summer months and during the autumn and winter hunt (Figure 2).

### 3.2 Calving, Juveniles, and Size

Two porpoises are reported as females with fetuses (OID: 7, 11). The two pregnant porpoises were reported in May and June in Umeå and Karlshamn, respectively (Figure 4C). Two additional porpoises were reported as potentially being a mother and calf pair (OID: 209, 214) near Hudiksvall and Örnsköldsvik, respectively (Figure 4C). One report from 1886 describes the annual porpoise hunt in Bramsnæs Bay, Denmark, that occurs from March to May, including that “many of the animals have a small cub in their belly but never more than one” (OID 60).

**FIGURE 3:**
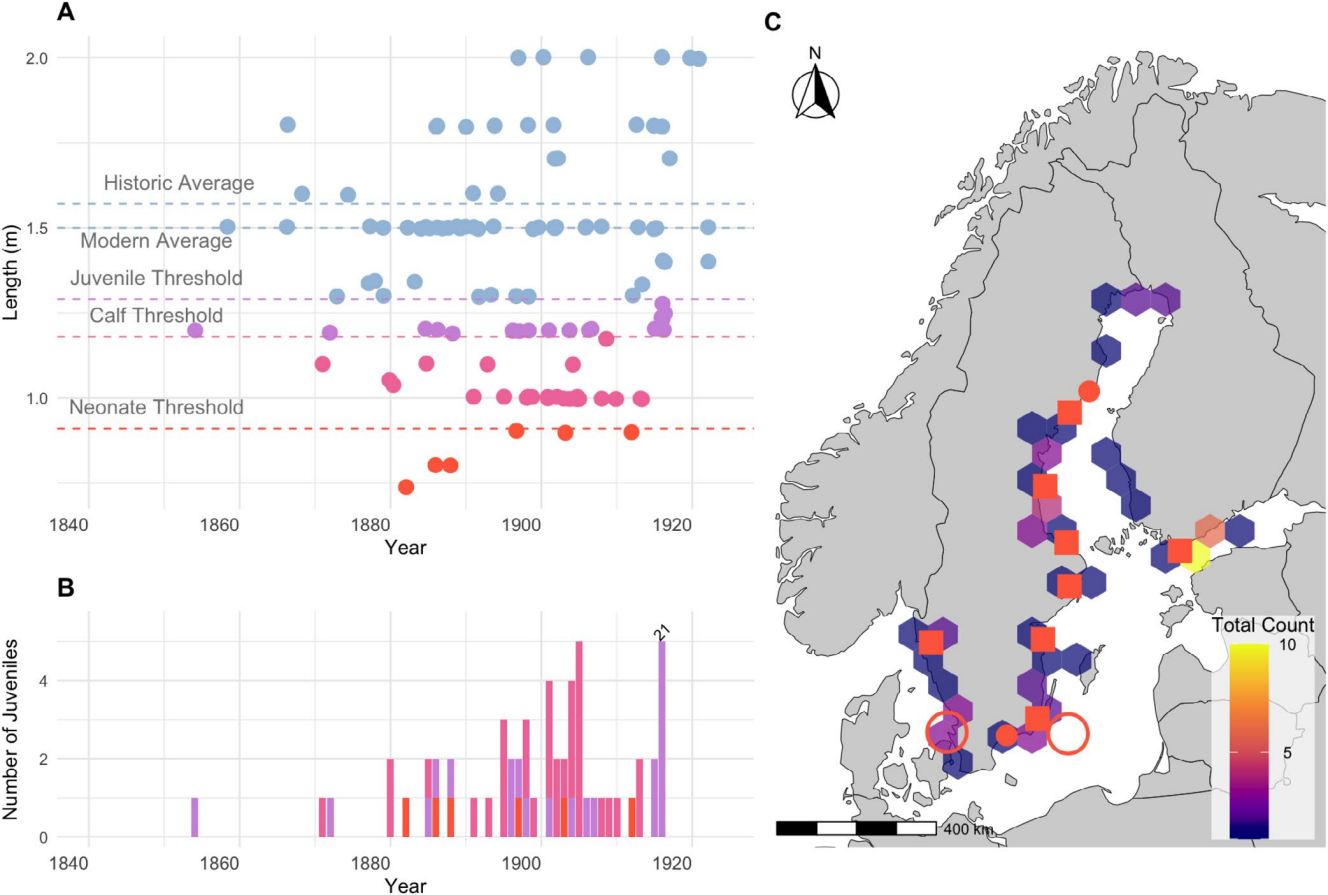
Juvenile harbour porpoises identified by length. (A) Length in meters recorded for harbour porpoises plotted over time. The dark blue dotted lines represent the average historic and modern length of non‐juvenile porpoises. (B) Time‐series plot of the sub‐adult harbour porpoises from 1870 to 1924. (C) Hexplot of sub‐adult porpoises for all periods. The red dots indicate reported pregnant porpoises, the red squares indicate neonates and mother‐calf pairs, and the circles indicate putative modern breeding grounds. Note that only ~12% of the total number of porpoises include length data. Juvenile counts are likely underestimates.

**FIGURE 4:**
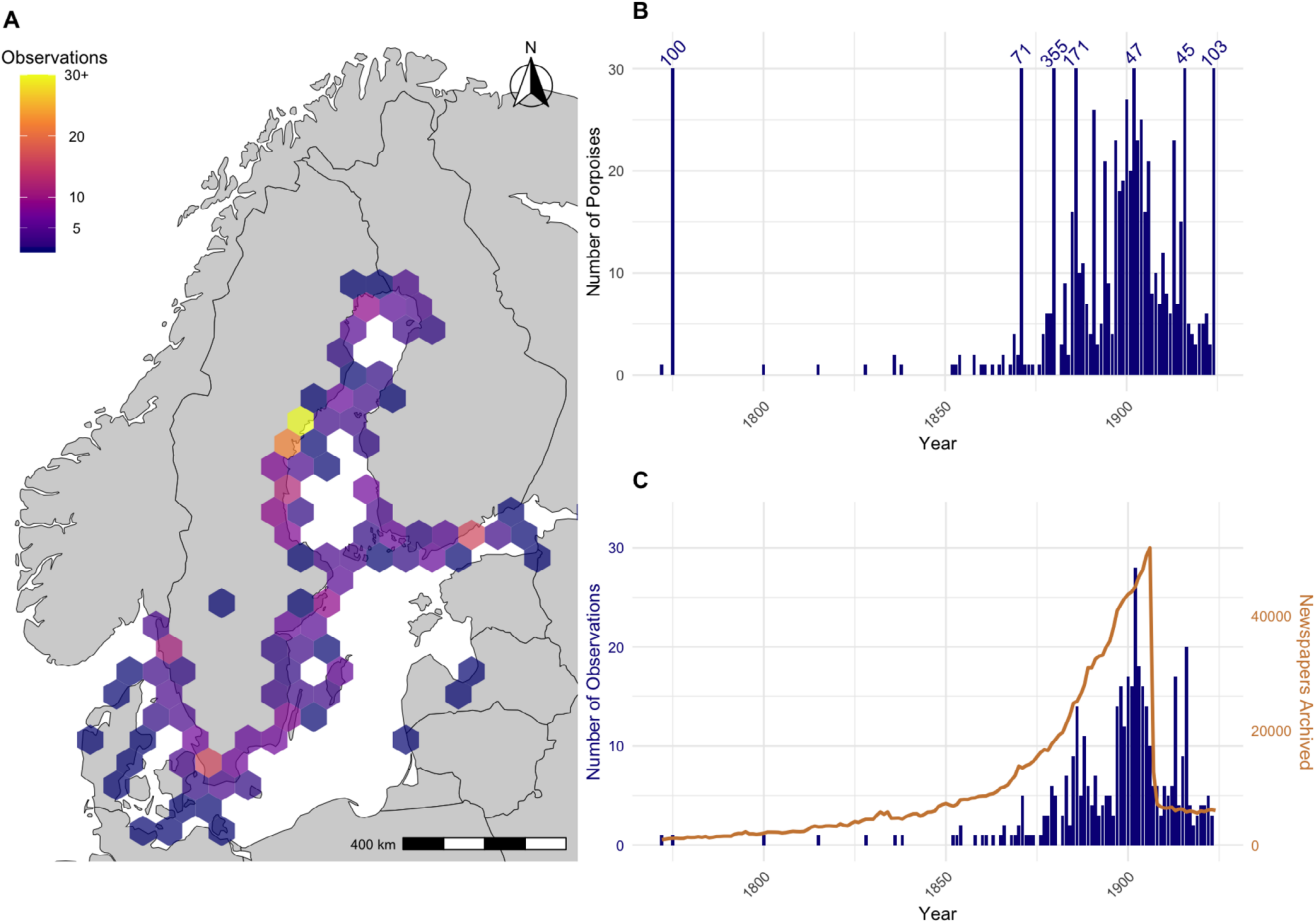
(A) The number of harbour porpoise observations (OIDs) plotted by location as a hex map. (B) Time series plot showing the number of porpoises reported over time. Note that one OID can contain more than one harbour porpoise. (C) Number of OIDs over time (blue, left axis) and number of newspapers archived in Sweden (orange, right axis).

Of the harbour porpoises for which there are lengths reported, approximately 43% (*n* = 52) are classified as sub‐adults. This includes six neonates (3.4%), 24 calves (13.6%), and 22 juveniles (12.5%; Figure 3). Additionally, ~57% (*n* = 68) are classified as adults (Figure 3). The first juvenile harbour porpoise was reported in 1854, and they were regularly reported starting in 1865 (Figure 4B). The juvenile harbour porpoises are reported along the majority of the Swedish coast and along the Finnish coast, including the northernmost part of the Bothnian Bay (Figure 4C).

The average length of porpoises in the “adult” age‐class (> 1.29 m) was 1.57 m (*n* = 68). This is slightly larger than the combined average length of modern adults in the study region. The historic average is greater than the modern male average (1.45 m) but falls slightly below the modern female average (1.6 m). Critically, the historic animals could not be classified by sex. Thus, the 1.29 m length cutoff used to define “adult” for the historic animals likely includes some juvenile females, whose length at physical maturity is generally higher (1.39 m). The inclusion of these smaller, sub‐adult females likely results in a slight underestimation of the true historic average adult length, suggesting the difference between historic and modern porpoises may be more pronounced than reported here. While the historical lengths are likely approximate and often reported in rounded figures, as is typical of historical, non‐scientific accounts, the relatively wide range of reported length measurements (0.7 m to 2 m) suggests that the data captures real variation in body size.

### 3.3 Impact of Archived Newspapers on Porpoise Observations

The number of archived newspapers reflects reporting opportunity and archiving rather than systematic observation or sampling effort. The number of Swedish newspapers archived decreases after 1907 as the National Library of Sweden Newspaper Archive shifts to documenting the main newspapers, reducing the number of newspaper titles archived from ~100 to less than 50 (Figure 3C). Spearman's test showed a strong influence between the number of newspapers archived and the number of OIDs (rho = 0.528, *p* = < 0.001) and a moderate influence between the number of newspapers and the number of harbour porpoises reported (rho = 0.438, *p* = < 0.001). The linear regression did not find a significant linear relationship between the number of newspapers and the number of porpoises (*β* = 3.26 × 10^−4^, *p* = 0.412), suggesting a linear model was not appropriate for the data. In contrast, the GAM revealed a significant nonlinear relationship (*χ* ^2^ = 859.8, *p* < 0.001), indicating that porpoise counts increased with newspaper availability but in a non‐linear manner.

The Mann–Whitney *U* test revealed a significant difference between the number of porpoises before and after 1900 (*W* = 241.5, *p* < 0.001). However, when accounting for differences in the number of newspapers archived in a Poisson GLM, the early 1900s period was associated with fewer harbour porpoise reports overall (*β* = −0.27, *p* < 0.001), despite the number of newspapers being a strong positive predictor of reported porpoise counts (*β* = 1.79 × 10^−5^, *p* < 0.001).

Sub‐regional Poisson GLMs show substantial spatial variation in the temporal trends. The Bothian Bay exhibited strong temporal trends, with porpoise counts nearly doubling in the early 1900s (*β* = 0.684, *p* < 0.001), largely independent of newspaper activity (*β* = 4.78 × 10^−6^, *p* = 0.395). The Bothnian Sea counts increased moderately over the same period (*β* = 0.429, *p* = 0.028), but the higher newspaper coverage likely caused the increase (*β* = 1.70 × 10^−5^, *p* = 0.004). The number of porpoises West of the Baltic decreased in the early 1900s (*β* = −1.51, *p* < 0.001), with little effect from the number of newspapers (*β* = 2.23 × 10^−6^, *p* = 0.421). In the Baltic Sea, the number of reported counts remained relatively stable (*β* = −0.255, *p* = 0.103), but the number of archived newspapers was negatively associated with porpoise reports (*β* = −2.09 × 10^−5^, *p* < 0.001). These results indicate that temporal trends in the number of porpoises differ by sub‐region, with some sub‐regions showing clear early‐1900s declines or increases while others remain stable. This reduction in archived newspapers corresponded to a slight decrease in porpoises reported (Figure 2B), though the larger newspapers occasionally reprint observations from non‐archived smaller newspapers.

## 4 Discussion

### 4.1 Presence and Distributions

The historic distribution of harbour porpoises in Swedish waters appears to be distinct from the modern one. In modern times, few animals have been observed north of the Åland Islands (HELCOM/ASCOBANS Harbour Porpoise database), and a northern management border has been proposed between 60.5° N at the Swedish coast and 61° N at the Finnish coast (ICES 2020). The historic range of the harbour porpoise, however, extends to the northernmost parts of the Gulf of Bothnia with higher concentrations (30+ individuals) along the Swedish coast between Hudiksvall and Örnsköldsvik. Their frequency in the Bothnian Bay, however, is not clear. Reports from the 1800s and 1890s suggest that porpoises are rare in the sub‐region (OID: 47, 54, 55, 115, 131, 331) and “should rarely be caught” (OID: 115). Conversely, their occurrence was noted as not unusual by 1899 and into the 1900s (OID: 337). In 1899, the newspaper Umeåbladet reported: “The presence of porpoises in our waters is far from as rare as one might think due to the information regarding the sea animal visible in the Sundsvall area.” This contrasts sharply with the contemporary spatial distribution of harbour porpoises in the SAMBAH project, which generally reported low predicted detection rates in the waters of 5–80 m depth from north of Gotland to the Åland and Archipelago Seas (Carlén et al. 2018). It is important to note that the SAMBAH acoustic monitoring did not survey deeper waters, which include considerable parts of the Baltic Proper, north and east of Gotland. Areas exceeding a 20% monthly probability of detection were restricted to the south and west of the Baltic Proper, though these highly important areas accounted for only ∼30% of all recorded detections (Carlén et al. 2018). National monitoring programs and the SAMBAH project have consistently identified seasonal movements of harbour porpoises within the Baltic Sea and west of the Baltic Sea (Benke et al. 2014; Carlén et al. 2018; Owen et al. 2021), characterized by an increased presence around the offshore banks east and southeast of Öland and Gotland in summer, and a more coastal distribution in winter. Specifically, analyses carried out in German waters have linked the winter distribution of the assumed Baltic Proper population to cold air temperatures and air temperatures lower than water surface temperatures (Gallus et al. 2012), which is interpreted as a migration pattern toward the southwest during winter months. Since the archival data primarily comes from Sweden and Finland, the absence of porpoises outside these countries' coastlines should not be interpreted as a historical absence, as their presence is not captured in the available records.

A historical presence of harbour porpoises in the Gulf of Bothnia is a key finding and suggests a substantial difference between the historic and modern distributions, corresponding to the modern absence of harbour porpoises from sub‐regions accounting for approximately one‐third of the historically documented range in the Baltic region. Range contractions, linked to environmental stressors and anthropogenic impacts, affect species distribution and, eventually, survival. It is especially concerning for marine mammals, which often rely on large, stable environments for migration, foraging, breeding, and social behaviors. The historical presence of harbour porpoises in the Gulf of Bothnia challenges the idea that the current range reflects ecological limits of the harbour porpoise in the Baltic Sea region. While the archival newspaper data allow for reconstruction of historic presence and relative spatial extent, they do not directly test the ecological systems producing the range retraction. Here, we integrate the historical patterns identified with insights from contemporary oceanographic and ecological studies in attempts to understand the changes observed. Several factors could contribute to the retraction of harbour porpoises to south of the Åland Islands, which can be broadly categorized into three interconnected factors: climatic and oceanographic factors, prey availability, and anthropogenic impact. These factors may be further exacerbated by demographic constraints inherent to a small population, creating a mechanism that actively drives the maintenance of a retracted range.

The Gulf of Bothnia is characterized by its low‐salinity brackish waters, a state that has existed since the Late Holocene (~3000 BP; Widerlund and Andersson 2011). This environment results in a sensitive ecosystem and significantly limits biodiversity as few species are capable of colonizing the sub‐region (Snoeijs‐Leijonmalm 2017). The lower salinity water favors brackish and even freshwater species over marine species, resulting in a shift in community composition and negatively impacting large‐bodied marine taxa, potentially affecting marine predators reliant on these species as prey (Rousi et al. 2013; Weigel et al. 2015; Mäkinen et al. 2017; Snoeijs‐Leijonmalm 2017). This makes the sub‐region a sensitive ecosystem. Harbour porpoises are known to visit areas of low salinity such as estuaries and rivers (Cucknell et al. 2020; Stern et al. 2017; Wenger and Koschinski 2012; Rekdahl et al. 2023; Koschinski 2001; Kesselring et al. 2017), and genomic analyses have suggested an adaptation to the salinity gradient (Celemín et al. 2023), suggesting that low salinity is not a physiological barrier. However, the brackish environment means the few adapted species are sensitive to change, making the Gulf of Bothnia's species‐poor ecosystem acutely vulnerable to disturbance, particularly the loss of prey, impacting the feasibility of the habitat.

Eutrophication, driven by nutrient runoff from expanding agriculture and urban centers, has had deleterious effects on benthic communities and has led to reduced biodiversity (Österblom et al. 2007; Rousi et al. 2013; Weigel et al. 2015). The severity of the nutrient over‐enrichment in the Gulf of Bothnia is debated (Andersen et al. 2011; Voss et al. 2011). The decline in benthic species has reduced the prey available for demersal fish and other predator species (Rousi et al. 2013; Mustamäki and Mattila 2015). Similarly, there has been a reduction in dissolved oxygen concentrations since the mid‐1950s, with a trend of negative 0.1 mL L^−1^ per decade in the Bothnian Bay (Polyakov et al. 2022). Deoxygenation of the sea has led to expanding areas of oxygen minimum zones and hypoxia, primarily linked to increased nutrient input (Carstensen et al. 2014). In 2023, 35% of the bottom of the Baltic Sea were anoxic or hypoxic (Hansson and Viktorsson 2024). These hypoxia/anoxic regions are less likely to be habitable for harbour porpoises, as prey will be less available (Almroth‐Rosell et al. 2021; Krapf et al. 2022; Koschinski et al. 2024). The weakening food web in the Bothnian Sea has been linked to overfishing and eutrophication (Faithfull and Bergström 2025).

Beyond these primary stressors, the extremely small population size may itself be a barrier to range expansion. If the remaining population of harbour porpoises is spread throughout its full historical range, the resulting low population density would likely induce the Allee effect. An Allee effect occurs when the positive benefits of groups, such as cooperative defense or improved mate finding, are lost as a population becomes too sparse, resulting in a correlation between population density and individual fitness (Courchamp et al. 1999; Walter et al. 2017). Essentially, the population growth rate declines at low population densities.

The Allee effect has been suggested in other marine mammal species, most notably relating to reproductive success due to low‐density populations. For example, the difficulty in finding a mate across a large habitat at extremely low population densities is proposed to be a factor limiting the recovery of the critically endangered North Atlantic right whale ( *Eubalaena glacialis* ; Caswell et al. 1999). A similar concern is driving population modeling for the polar bear ( *Ursus maritimus* ), where low population densities, unpredictable habitat, and harvest‐depleted male populations lead to infrequent mating encounters (Molnár et al. 2008, 2014). Evidence for an Allee effect was also suggested during the early stages of recovery from overexploitation in the Antarctic Fur Seal ( *Arctocephalus gazella* ; Nagel et al. 2021).

In the case of the Baltic proper harbour porpoise, a potential Allee effect could incentivize individuals to stay in the core, higher‐density area for mating, thus perpetuating a range retraction that was caused by an initial stressor. This low‐density scenario suggests that the costs of mandatory seasonal migrations south to avoid ice‐covered water may no longer be justified. Instead, the need for reproductive assurance, likely concentrated in the core southern part of the Baltic, outweighs the benefits of returning to the now‐peripheral, low‐density habitat, essentially incentivizing a maintained range retraction. Thus, while not being the initial driver of habitat retraction, a component Allee effect on the much‐reduced population size may be a factor keeping the population in a smaller range.

### 4.2 Population Size

Zooarchaeological analysis suggests that harbour porpoises were once more abundant in the southern Baltic Sea region (Sommer et al. 2008; Lõugas and Bērziņš 2023; van den Hurk and Aiken 2025). Medieval texts further support a sizable harbour porpoise population in the Baltic Sea region (van den Hurk and Aiken 2025). 18th‐ and 19th‐century historical documents provide evidence of harbour porpoises present in Swedish waters (von Linné et al. 1792; Flower et al. 1866), including a report of a “whale and salmon fishery” in northern Gotland (Caddy 1887). Historical data from bounty projects, bycatch records, and observations of dead stranded animals show that harbour porpoises were numerous in the Baltic and Gulf of Bothnia during the first half of the 20th century (Johansen 1929; Lönnberg 1940; Tägström 1940; Psuty 2013; HELCOM 2023). Current estimates, however, suggest a small Baltic Proper harbour porpoise population of around 500 individuals concentrated in the southwestern Baltic (Amundin et al. 2022) and a larger but declining Belt Sea population of around 14,000 individuals (Gilles et al. 2023). Harbour porpoises in the Baltic region are comprised of two morphologically (Börjesson and Berggren 1997; Huggenberger et al. 2002; Galatius and Kinze 2012) and genetically (Wiemann et al. 2010; Celemín et al. 2023; Autenrieth et al. 2024) distinct populations: the Baltic Proper and the Belt Sea population. While these populations are considered distinct, their overlapping range creates a complex dynamic for conservation and population management. Differentiation of these populations relies on genomic identification or geometric morphometric analysis of skeletal material (Galatius and Kinze 2012; Celemín et al. 2023); thus, differentiation based on visual observation is not possible. However, harbour porpoises equipped with satellite tags within the Belt Sea assessment unit are rarely registered to move farther east than 15.0° E (Sveegaard et al. 2015), which is around the Danish island of Bornholm, suggesting a modern spatial divide between the populations. The harbour porpoises included in the archival newspaper accounts are likely a mix of Belt Sea and Baltic Proper individuals. While historic documents cannot provide quantitative estimates of population size, the number of observations is greater than what is observed in modern‐day, suggesting that the historic population size was larger.

Anecdotal newspaper reports indicate an increase in harbour porpoise observations in the Bothnian Sea during the early 1900s. Several newspapers described “a mass immigration” of porpoises into the region, often highlighting perceived negative impacts on fisheries. Our data confirm an increase in porpoise observations in the Bothnian Bay and Sea, but not in the Baltic Sea or west of the Baltic. While historical records report concern about interactions with fisheries, modern ecological studies indicate that harbour porpoises and other top predators can coexist with sustainably managed fisheries, providing important ecosystem services and regulatory functions in aquatic ecosystems (Woodstock et al. 2025; Kiszka et al. 2022). Newspaper records from the 1920s show no evidence of a population decline, suggesting that the reduction occurred later in the 20th century. Although newspaper observations provide some indication of relative abundance, they should be interpreted cautiously as a proxy for true population size. Surveys beginning in the 1950s documented a significant decrease in harbour porpoise numbers (Berggren and Arrhenius 1995), but the absence of consistent data between the 1920s and 1950s means that the exact timing of the onset of the decline remains uncertain.

### 4.3 Calving, Juveniles, and Size

Female harbour porpoises give birth to a single calf every year (or every other year) once reaching sexual maturity at 3–5 years of age (Lockyer and Kinze 2003; Kesselring et al. 2017). Currently in the Baltic region, mating mainly occurs from July to August, with calves subsequently born from May to August after an approximately 10‐month gestation period (Börjesson and Read 2003; Lockyer and Kinze 2003). The three individuals reported as pregnant were reported in May and June, fitting with modern calving timing. OID 7 and 11 had size descriptions (40 cm and 4.6 kg, respectively) of the fetuses.

While Belt Sea porpoises are often observed around Kullen (Sweden) in the Kattegat during the reproductive season (Sveegaard, Teilmann, Berggren, et al. 2011; Sveegaard, Teilmann, Tougaard, et al. 2011; Teilmann et al. 2022; Stedt et al. 2023), the location(s) of calving are less clear for the Baltic Proper population, though it is thought to occur around Hoburgs bank and Midsjöbankarna (Figure 4C). When considering the porpoises assigned to the age category neonates, as well as the reported mother‐calf pairings and pregnant porpoises, there is a distribution as far north as the northernmost part of the Bothian Sea. The range of juvenile harbour porpoises largely mirrors that of the whole distribution, suggesting that juvenile harbour porpoises occur in the same places as adult harbour porpoises.

### 4.4 Archived Newspapers and Porpoises

Porpoise records are influenced by reporting effort and media availability. As such, the historic newspaper reports do not represent systematic sampling and are instead shaped by anthropogenic choices, including editorial practices, cultural interest, and archival processes. While newspaper availability is a precondition for reporting and subsequent identification of historic harbour porpoise observations, it is not a measure of observational effort. The value in these analyses lies not in treating newspapers as a proxy for sampling effort, but in demonstrating that porpoise reporting exhibits strong, structured relationships with newspaper archiving despite the absence of systemic observation. The statistical structure in a non‐systematic dataset indicates that porpoise reporting is not arbitrary, but reflects underlying socio‐ecological processes.

### 4.5 Future Directions

Together, these data suggest that the current population distribution is not reflective of the species' potential range. The historic period around the mid‐20th century appears to be a critical time for the modern population. Looking forward, the potential for increased abundance through successful conservation efforts, combined with warming ocean trends due to climate change, suggests porpoises in the Baltic region may attempt to reoccupy their historic northern range, at least during spring–summer–fall when sea ice is absent; however, ecological heterogeneity between the Baltic Proper and the Bothnian Sea and Bay indicates that porpoises are unlikely to occur there year‐round, either historically or in the future. Nonetheless, as a consequence of continued warming, harbour porpoises could eventually return to their former northern range, a trend observed in other marine species (Stafford et al. 2022; Coulon et al. 2024; for exceptions, see Le Luherne et al. 2024) and marine mammals (Hamilton et al. 2019; Lameris et al. 2021; Stafford et al. 2022).

While recolonization remains speculative and contingent on future ecological conditions, the historical evidence demonstrates that these sub‐regions were previously in the range of harbour porpoise distribution. As such, potential recolonization should be considered in conservation, particularly in the context of successful population recovery and ongoing climate‐driven environmental change. The historic retraction of harbour porpoises from northern Baltic waters likely reflects, at least in part, aggregate anthropogenic pressures rather than a fixed anthropogenic limit, and conservation and management strategies should not assume that the current distribution is the natural or baseline. Proactive protection and spatial management with ecosystem‐based fisheries management are warranted to help ensure that future recovery is not constrained by legacy impacts or ongoing human pressures in these regions.

The anthropogenic pressures implicated in the range retraction of harbour porpoises in the Bothnian remain present today. Prey availability and habitat conditions may currently be insufficient to support substantial (re)colonization (Pekcan‐Hekim et al. 2016). Consequently, the ongoing nature of these stressors may continue to limit successful re‐colonization in the absence of strong conservation and mitigation measures. Therefore, proactive protection and spatial management with ecosystem‐based fisheries management in these historic northern sub‐regions are essential for future conservation efforts. This necessitates future ecological and genomic analyses to precisely examine past population size and changing environments, which will ultimately inform minimum viable population targets and future distribution scenarios for the region's harbour porpoise populations.

## 5 Conclusions

Through the lens of MHE, using data collected from Swedish archival newspapers between the late 18th and early 20th centuries, this study established a crucial time‐depth for the conservation of harbour porpoises in the Baltic Sea region. Our findings provide compelling evidence of a substantially different historical population structure and distribution, with three key implications for conservation:

1. Range Retraction: We demonstrate a notable range retraction, suggesting the loss of approximately a third of the historic range in the Gulf of Bothnia. Historical sighting patterns confirm the seasonal utility of these areas, with reports of porpoises west of the Baltic Sea (or approximately the Belt Sea assessment unit) primarily from April to November, the Baltic Sea from January to August, and the Bothnian Sea and Bay in the summer. Several likely interconnected mechanisms have been proposed in the literature that could explain the cause of the range retraction, including the availability of prey potentially reinforced by an Allee effect.
2. Larger Historical Population Size: Historical observation patterns indicate a larger historical population size. Harbour porpoises were reported with higher frequency than seen today, and key indicators of reproduction were observed across the historic range: sub‐adults were present alongside adults and pregnant females were identified substantially farther north than currently observed.
3. Utility of Historical Sources: Finally, the utility of historical sources in defining the former range and robustness of harbour porpoises in the Baltic region is shown. While historic newspapers are subject to selection and reporting biases, preferentially reporting “interesting” and extraordinary events, the temporal consistency and wide geographic coherence of the observations validate the findings and enable the contextualization of modern population dynamics.

Looking ahead, the historical evidence highlights that the current distribution does not reflect the species' full range, and conservation strategies should not assume that the present range represents a natural baseline. Proactive protection and management will be essential to ensure that future population recovery is not stymied by legacy impacts or ongoing anthropogenic pressures.

## Author Contributions

**M. Aiken:** conceptualization (equal), data curation (lead), formal analysis (lead), methodology (equal), visualization (lead), writing – original draft (lead), writing – review and editing (lead). **P. Erikson:** formal analysis (supporting), project administration (equal), supervision (supporting), writing – review and editing (supporting). **C. Kinze:** writing – review and editing (equal). **J. Carlström:** conceptualization (lead), data curation (equal), funding acquisition (lead), project administration (equal), supervision (lead), writing – review and editing (equal).

## Funding

This work was supported by Carl Tryggers Stiftelse för Vetenskaplig Forskning, CTS22:2074. Havs‐och Vattenmyndigheten, 2024‐001531.

## Conflicts of Interest

The authors declare no conflicts of interest.

## Supporting information


**Table S1:** Table with harbour porpoise observation IDs (OIDs) and report IDs (RIDs) (see supplementary documents).

## Data Availability

All of the newspapers referenced in this paper are publicly available through the Swedish Royal Library (https://tidningar.kb.se/). The curated dataset is available in the supplementary of the paper.

## Appendix A

### Archival Search Strategy and Data Screening

To assess the sensitivity of our search strategy, we tested additional search terms and spelling variants commonly associated with harbour porpoises in historical sources, including archaic forms and other related terms. For example, spelling variants “phocoena,” “phocaena,” and “phocana” were used as well as antiquated binomials “communis” and “delphinus.” Search terms were kept even when their results were highly redundant with other search terms in attempts to identify as many reports as possible. An exception is the term “delphin,” which was tested but predominantly returned references to ships or vessel names rather than cetaceans, contributing mostly noise with no identified unique records. To identify additional search terms, we consulted the Svenska Akademiens Ordbokas (SAOB, Swedish Academic Dictionary), which provides three additional terms: “isa,” “springare,” and “tumlett”. Isa, also mentioned in newspapers as a way coastal communities would call small cetaceans, was excluded as a search term as it is a verb that means “to ice” or “to freeze” as well as being a first name. “Springare,” which is now used to refer to common dolphins ( *Delphinus delphis* ), may have been used in the past to refer to the harbour porpoise. It also means “steed” and “knight” and as such, is used for horses as well as chess. Like “delphin,” no unique observations were identified for “isa,” “springare” or “tumlett”. Below is a flow chart that shows the total number of archived newspapers, the search terms, and the filtering used.

## Appendix B

### Data

**TABLE B1:** Maximum and minimum weights and length for cetacean species used for making exclusion criteria, including harbour porpoise (Pp) (Christina Lockyer and Kinze 2003) and dolphin species known to visit the Baltic region: Common dolphin (Dd) (Perrin 2018), bottlenose dolphin (Tt) (Wellsa and Scott 2024), white‐beaked sided dolphin (La) (Carl Christian Kinze 2009), and neonate orcas (Oo) (Perrin 2014).

Species	Min weight	Max weight	Min length	Max length
Pp		89 kg		1.9 m
Dd	70 kg	230 kg	1 m	2.3 m
Tt	150 kg	200 kg	2 m	4 m
La	180 kg	360 kg	2.4 m	3.1 m
Oo	200 kg		2 m	2.5 m

**TABLE B2:** Age‐length classification categories for porpoises used to analyze historic data, adapted from the established criteria (Lockyer 1995; van Elk et al. 2019; Neimanis et al. 2022) to account for sex‐specific maturation lengths in the absence of individual sex data; the male adult length (> 1.29 m) was used.

Age classification	Length range (m) used for historic harbour porpoises	Sex based	
		Male (m)	Female (m)
Neonate	≤ 0.91	< 0.91	
Calf	> 0.91 to ≤ 1.17	> 0.91 to ≤ 1.17	
Juvenile	> 1.17 to ≤ 1.29	> 1.17 to ≤ 1.29	> 1.17 to ≤ 1.39
Adult	> 1.29	> 1.29	> 1.39
